# The Antioxidant Activity of *Undaria pinnatifida* Sporophyll Extract Obtained Using Ultrasonication: A Focus on Crude Polysaccharide Extraction Using Ethanol Precipitation

**DOI:** 10.3390/antiox12111904

**Published:** 2023-10-24

**Authors:** Jae-Hoon Lee, Jeong-Heon Kim, Se-Myung Kim, Jae-Yong Kim, Jae-Hoon Kim, Su-Jin Eom, Min-Cheol Kang, Kyung-Mo Song

**Affiliations:** 1 Research Group of Food Processing, Korea Food Research Institute, Wanju 55365, Republic of Korea; l.jaehoon@kfri.re.kr (J.-H.L.); k.jeongheon@kfri.re.kr (J.-H.K.); jy_kim@kfri.re.kr (J.-Y.K.); k.jaehoon@kfri.re.kr (J.-H.K.); eom.su-jin@kfri.re.kr (S.-J.E.); 2 Greating Laboratory, Hyundai Green Food Ltd., Yongin 16827, Republic of Korea; semyungkim@hyundaigreenfood.com

**Keywords:** *Undaria pinnatifida* sporophyll, ultrasonication, antioxidant activity, polysaccharide

## Abstract

*Undaria pinnatifida*, a marine biological resource from which antioxidants such as polysaccharides can be obtained, is primarily distributed in the coastal areas of East Asia. Reactive oxygen species (ROS) are essential for physiological processes; however, excess ROS levels in the body result in cellular oxidative damage. Several extraction methods exist; however, factors such as long extraction times and high temperatures degrade polysaccharides. Therefore, this study aimed to increase the yield of *U. pinnatifida* sporophyll extract (UPE), a *U. pinnatifida* byproduct, using ultrasonication, an environmentally friendly extraction method, and identify UPE components with antioxidant activity. UPE_2, 4, 6, and 8 extracts were obtained at extraction times of 2, 4, 6, and 8 h, respectively. UPE_8 had the highest yield (31.91%) and polysaccharide (69.22%), polyphenol, (8.59 GAE μg/mg), and fucoxanthin contents (2.3 μg/g). UPE_8 showed the greatest protective and inhibitory effects on ROS generation in H_2_O_2_-damaged Vero cells. Ethanol precipitation of UPE_8 confirmed that UPE_8P (precipitate) had superior antioxidant activity in Vero cells compared to UPE_8S (supernatant). UPE_8P contained a large amount of polysaccharides, a major contributor to the antioxidant activity of UPE_8. This study shows that UPE_8 obtained using ultrasonication can be a functional food ingredient with excellent antioxidant activity.

## 1. Introduction

Reactive oxygen species (ROS), a family of active molecules, are products of the respiratory process and are essential for physiological processes [1]. Under normal conditions, ROS production is regulated by the antioxidant defense system of the body [2]. However, pathological conditions induced by environmental influences, stress, and aging can lead to ROS regulation failure, resulting in oxidative damage to cellular macromolecules [3,4]. This oxidative damage accelerates age-related diseases such as cancer, inflammation, and cardiovascular diseases [5,6]. Therefore, studies on antioxidants that can effectively scavenge ROS are being conducted, with a focus on antioxidants obtained from natural products such as vitamins, carotenoids, and polysaccharides [3,7,8].

*Undaria pinnatifida* is a marine biological resource from which polysaccharides can be obtained, and it has traditionally been used as a therapeutically active substance for edema, phlegm removal, and diuresis [9]. *U. pinnatifida* sporophyll is a byproduct of *U. pinnatifida* and contains various bioactive compounds, including polysaccharides, polyphenols, polyunsaturated fatty acids, vitamins, and carotenoids [10,11,12]. Increasing research is being conducted on extraction methods targeting bioactive compounds, which have shown diverse biofunctional activities [13,14,15]. Among these methods, the hot or boiling water extraction method is commonly used owing to its simplicity and capacity to extract large quantities [16]. However, a high extraction temperature may lead to the decomposition of bioactive compounds, resulting in a low extraction yield. In addition, extraction methods using various solvents and extraction methods using enzymes are also being investigated [17,18]. While these methods have the advantage of high extraction purity, their industrial application is limited by their requirement of an additional process to remove residual solvents and the use of expensive enzymes.

Ultrasonic extraction is a safe and environment-friendly extraction technology that allows for large-scale extraction with the advantages of high yield, low temperature, short extraction time, and low cost [19,20,21]. Ultrasonication releases a large amount of energy into the liquid phase, causing the collapse of the cell walls in the organic materials, thereby allowing the solvent to effectively enter the cell and resulting in effective extraction [22,23]. Ultrasonication is also highly industrially applicable as it is easy to scale up to large volumes [24], suggesting a promising outlook for the industrial application of *U. pinnatifida* sporophyll extract using this method. In addition, an ethanol precipitation method is widely used to separate crude polysaccharides from the active ingredients of the extract [25,26]. This extraction technique relies on the principle that polysaccharides present in the extract precipitate upon treatment with ethanol. Moreover, this method has been widely used to produce crude polysaccharides in various studies [25,26], including those investigating changes in polysaccharide extraction efficiency and the antioxidant activity of polysaccharides depending on ethanol concentration.

In our previous study, we confirmed the effect of the solvent on extraction efficiency during *U. pinnatifida* sporophyll extraction [13,19]. In particular, in large-scale extraction experiments for industrialization, we found that extraction using water was the most effective in terms of yield [19]. Furthermore, by conducting preliminary research, we determined the optimal conditions as 1080 W power, 80% amplitude, 20 kHz frequency, and a 30 °C extraction temperature. Therefore, in the present study, we aimed to increase the yield of sporophyll by conducting ultrasonic extraction using water. We prepared different ultrasonication extracts by varying the extraction time and analyzed their antioxidant activity in Vero cells. In addition, to identify the components contributing to the antioxidant activity of the *U. pinnatifida* sporophyll extract, we extracted polysaccharides using ethanol precipitation, determined their proximate composition and molecular weight, and analyzed the constituent monosaccharides and antioxidant activity.

## 2. Materials and Methods

### 2.1. Ultrasonic Extraction and Ethanol Precipitation

**Figure 1:**
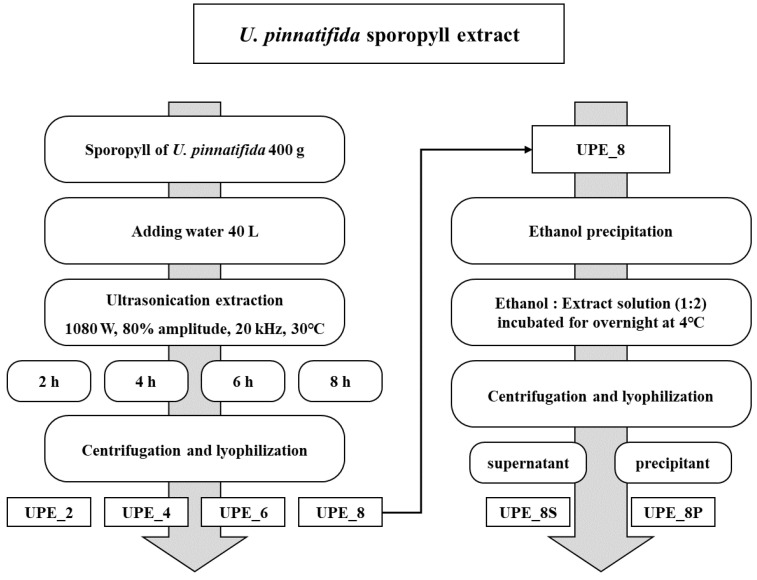
Schematic of *U. pinnatifida* sporophyll ultrasonication extraction.

*U. pinnatifida* sporophyll was obtained from a local market in Wando (Jeollanam-do, Korea). The sporophyll was powdered using a blender after drying (Hanil, Seoul, Republic of Korea). Powdered *U. pinnatifida* sporophyll (400 g) was mixed with 40 L of water. The sporophyll was extracted using an MX Sonic (MX-12S2, Mirae Ultrasonic Tech., Bucheon, Republic of Korea) under the following conditions: 1080 W, 80% amplitude, 20 kHz, 30 °C, and four different extraction times (2, 4, 6, and 8 h) (Figure 1). In this study, other extraction conditions except time were determined by consulting a previous study [19]. After extraction, the extract was centrifuged (2700× *g*, 15 min, 4 °C) to obtain the supernatant, which was subsequently freeze-dried. The extracts were labeled UPE_2, UPE_4, UPE_6, and UPE_8 according to the extraction time of 2, 4, 6, and 8 h, respectively.

Ethanol precipitation was performed to separate the crude polysaccharides from the UPE (Figure 1). The reaction was performed overnight at 4 °C after mixing the UPE solution and 99.5% ethanol in a ratio of 2:1. The mixture was then centrifuged (12,000× *g*, 10 min, 4 °C) to obtain a supernatant and precipitate, which were subsequently freeze-dried. The supernatant and precipitate obtained were labeled UPE_8S and UPE_8P, respectively.

### 2.2. Polysaccharide, Polyphenol, and Fucoxanthin Content Analysis

The polysaccharide content of the UPEs was determined using the phenol–sulfuric acid method [13]. UPEs dissolved in distilled water were placed in a tube and mixed with a 5% phenol solution. After vortexing, sulfuric acid was added to the mixture and cooled to 25 °C. The absorbance of the mixture was measured at 470 nm, and the total polysaccharide content was calculated using glucose as the standard.

The polyphenol content of the UPEs was determined using the Folin–Ciocalteu method [3]. UPEs were mixed with a Folin–Ciocalteu reagent (Sigma-Aldrich, St. Louis, MO, USA). Next, 7.5% sodium carbonate solution was added to each sample, and the samples were then incubated for 2 h in the dark. The absorbance of the mixture was measured at 750 nm, and the total polyphenol content was calculated and expressed as μg gallic acid equivalents (GAE)/mg of sample.

The fucoxanthin content of the UPEs was determined using high-performance liquid chromatography (HPLC, Dionex, Sunnyvale, CA, USA) with an Inertsil ODS-3 column (4.6 × 250 mm, 5 μm) (GL Science, Tokyo, Japan) at 30 °C. All UPE samples were injected at a volume of 10 μL and eluted isocratically with 75% acetonitrile at a flow rate of 1 mL/min. The eluted peaks were detected using a UV detector at 450 nm, and qualitative and quantitative analyses were carried out using fucoxanthin standards [19].

### 2.3. Molecular Weight and Monosaccharide Composition Analysis Using High-Performance Liquid Chromatography

An HPLC system (Agilent 1100; Agilent Technologies, Santa Clara, CA, USA) coupled with an evaporative light scattering detector (Agilent) was used. Shodex OHPak SB-804HQ (8 × 300 mm, 10 μm particle size, Showa Denko, Tokyo, Japan) and SB-802.5 HQ (8 × 300 mm, 6 μm particle size) columns were used. All UPE samples (2 mg/mL in deionized water) were filtered using a 0.45 μm syringe filter, and the filtered samples were introduced into a column. The injection volume was 100 μL, and the flow rate was 0.6 mL/min. Peaks were detected using an RI detector, and the molecular weight was calculated using pullulan standards (Shodex standard P-82; Showa Denko).

For the UPE monosaccharide composition analysis, high-performance anion-exchange chromatography coupled with pulsed amperometric detection (Dionex, Sunnyvale, CA, USA) was used. A CarboPac™ PA10 column (2 mm × 250 mm, 10 μm particle size) was used for separation. All UPE samples (2 mg/mL) were hydrolyzed with trifluoroacetic acid and then introduced into a column. The injection volume was 20 μL, and the flow rate (1.0 mL/min) of the eluent was set under 18 mM NaOH/200 mM NaOH.

### 2.4. Cell Culture and Cell Viability

Vero cells, monkey kidney cells, were obtained from the American Type Culture Collection (ATCC, Rockville, MD, USA). The cells were cultured in Dulbecco’s modified Eagle medium (Gibco, Grand Island, NY, USA) containing 10% fetal bovine serum (Gibco) and 1% penicillin–streptomycin solution (Gibco) at 37 °C in a 5% CO_2_ humidified incubator.

An MTT assay was performed as previously described [27], with minor modifications to determine the effects of UPE on Vero cell viability. Briefly, Vero cells were seeded in a 96-well plate at a density of 1 × 10^4^ cells/well and incubated for 24 h. Various UPE and hydrogen peroxide (H_2_O_2_) concentrations were added to each well. After an additional 24 h of incubation, an MTT solution (Sigma-Aldrich) was added to each well, and the reaction proceeded for 3 h. The medium was removed, and DMSO was added to each well to dissolve the colored formazan crystals. The absorbance of the mixture was measured at 570 nm using a microplate reader. Cell viability was calculated based on the absorbance of the medium-treated group as a control.

### 2.5. Intracellular ROS Measurement

A DCFH-DA fluorescence assay was used to confirm the effect of UPE on ROS production in Vero cells [28]. Vero cells were seeded into a 96-well plate at a density of 1 × 10^4^ cells/well and incubated for 24 h. Various UPE concentrations were added to each well and incubated for an additional 1 h. After 1 h of incubation, 10 μM of DCFH-DA fluorescent solution (Sigma-Aldrich) was added to each well for 30 min. After replacing the medium with fresh medium, 1 mM H_2_O_2_ was added to each well, and the reaction proceeded for 30 min. The fluorescence intensity was measured at a wavelength of 485 nm (excitation)/535 nm (emission) using a microplate reader.

### 2.6. Western Blot Analysis

Vero cells (1 × 10^6^ cells/dish) were seeded on a 60-mm dish and incubated for 24 h. UPE_8P-treated cells were then treated with 1 mM H_2_O_2_. After 2 h, total protein was extracted using RIPA buffer containing a protease and phosphatase inhibitor cocktail (Thermo Fisher Scientific, Waltham, MA, USA). The protein content was determined using a bicinchoninic acid (BCA) assay (Pierce™ BCA Protein Assay Kit, Thermo Fisher Scientific). Thirty micrograms of protein from each sample were separated using a 12% sodium dodecyl-sulfate polyacrylamide gel electrophoresis gel (Bio-Rad Laboratories, Hercules, CA, USA) and transferred to polyvinylidene fluoride membranes (Bio-Rad Laboratories). The membranes were blocked with blocking buffer (EveryBlot Blocking Buffer, Bio-Rad Laboratories) and incubated with the corresponding primary antibodies (Bax, anti-rabbit, 1:1000 dilution; Bcl-2, anti-rabbit, 1:1000 dilution; β-actin, anti-rabbit, 1:2000 dilution; Cell Signaling Technology, Danvers, MA, USA) overnight at 4 °C. The membranes were washed with Tris-buffered saline containing Tween (TBS-T) and incubated with anti-rabbit secondary antibodies (HRP-conjugated antibodies, 1:5000; Cell Signaling Technology) for 2 h at room temperature. After a secondary wash with TBS-T, the bands were detected using an ECL reagent and observed using ChemiDoc™ XRS + (Image Lab™ Software ver. 6.1, Bio-Rad Laboratories).

### 2.7. Statistical Analysis

Data are presented as mean ± standard deviation from triplicate experiments. Statistical analysis was performed using SPSS Statistics version 20 (IBM Corp., Armonk, NY, USA). One-way analysis of variance (ANOVA) followed by Duncan’s test (*p* < 0.05) was used to measure the significance of the differences among multiple samples.

## 3. Results and Discussion

### 3.1. Extraction Yield, Polysaccharide, Polyphenol, and Fucoxanthin Content According to Extraction Time

Ultrasonication is an eco-friendly extraction method used in various studies to extract functional substances, and it has a high potential for use as a food additive as it does not involve the use of organic solvents during extraction [13]. In the present study, we prepared extracts according to different ultrasonication extraction times (2, 4, 6, and 8 h) and analyzed and compared the extraction yield, total polysaccharide, polyphenol, and fucoxanthin contents of the extracts. These *U. pinnatifida* sporophyll extracts, obtained using ultrasonication, showed excellent antioxidant activity (Figure 1).

**Figure 2:**
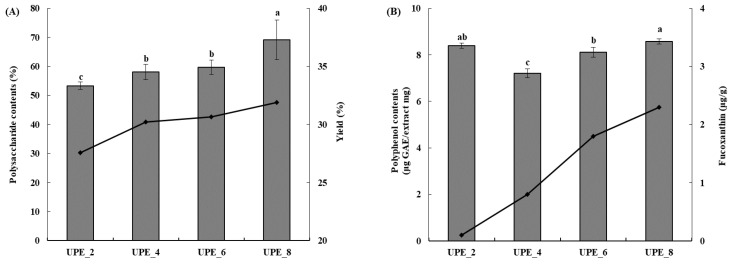
Extraction yield (black line) and polysaccharide content (gray bar) (**A**) and fucoxanthin content (black line) and polyphenol content (gray bar) (**B**) of UPEs according to extraction time. Values are expressed as mean ± standard deviation. Different letters (a–c) indicate significant differences found using one-way ANOVA followed by Duncan’s multiple range test (*p* < 0.05).

The extraction yield reportedly increases with extraction time in various methods, including ultrasonic extraction [29]. Our results showed that the extraction yield increased with increasing extraction time (Figure 2A). The lowest yield was 27.55% (UPE_2), increasing to 30.22% (UPE_4), 30.67% (UPE_6), and 31.91% (UPE_8). Therefore, the highest yield was achieved when ultrasonic extraction was performed for 8 h (UPE_8). Moreover, the total polysaccharide content of the UPE according to the extraction time was measured using the phenol–sulfuric acid method (Figure 2A). The total polysaccharide content increased as the extraction time increased (*p* < 0.05), similarly to the extraction yield. UPE_2, which was extracted for 2 h, showed a total polysaccharide content of 53.33%, and the total polysaccharide content increased as the extraction time increased, with UPE_8 showing the highest total polysaccharide content (69.22%, *p* < 0.05). In summary, the extraction yield and total polysaccharide content were the highest in the UPE extracted using ultrasonication for 8 h (UPE_8). Notably, UPE_8 showed no significant difference in yield and total polysaccharide content compared to the 24 h extract.

The polyphenol content was measured using the Folin–Ciocalteu method (Figure 2B). UPE_4, the 4 h extract, showed the lowest polyphenol content (7.21 GAE μg/mg, *p* < 0.05), and as the extraction time increased, the polyphenol content also increased, with the 8 h extract (UPE_8) showing the highest content (8.59 GAE μg/mg, *p* < 0.05). The fucoxanthin content was measured using HPLC (Figure 2B) and increased with extraction time, with 0.1, 0.8, 1.8, and 2.3 μg/g for UPE_2, UPE_4, UPE_6, and UPE_8, respectively.

Generally, yield is a primary factor determining the economics of an extraction method [30]. Additionally, seaweed polysaccharide content is important, with various studies showing that polysaccharides are the active ingredients responsible for the biofunctional activity of seaweed extracts [27,31]. Polysaccharides obtained from *Sargassum autumnale* (brown seaweed) exhibit antioxidant activity [27], and fucoidan, a sulfated polysaccharide extracted from New Zealand brown seaweed, effectively inhibits the growth of breast cancer cell lines [31]. Moreover, polyphenols and fucoxanthin are active ingredients in seaweed that are associated with antioxidant activity [19,27]: the higher the content of these ingredients, the higher the antioxidant activity [13,19,27].

### 3.2. Effect of UPE on Cell Viability and H_2_O_2_-Induced ROS Production in Vero Cells

**Figure 3:**
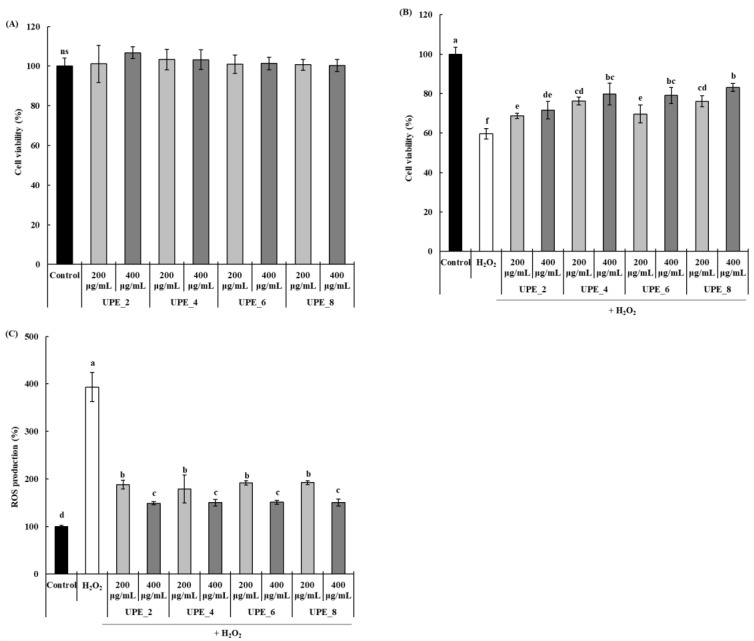
Effects of UPEs on Vero cell viability (**A**) and the protective effect against H_2_O_2_ (**B**) and ROS generation (**C**) of Vero cells induced with 1 mM H_2_O_2_. Values are expressed as mean ± standard deviation. Different letters (a–f) indicate significant differences found using one-way ANOVA followed by Duncan’s multiple range test (*p* < 0.05). ns: no significant difference between samples (*p* > 0.05). Control: medium-only treated group.

The antioxidant activity of the UPEs was confirmed by measuring the protective effect of UPEs in Vero cells exposed to H_2_O_2_. None of the UPEs affected the viability of Vero cells at any concentration, regardless of the extraction time (Figure 3A). To induce oxidative stress, cells were exposed to H_2_O_2_, and the protective effect of UPEs was assessed (Figure 3B). After exposure to H_2_O_2,_ the viability of Vero cells decreased significantly to 59.60% (*p* < 0.05), whereas it increased significantly after treatment with UPEs (*p* < 0.05). Notably, the cell protective effect increased with extraction time; treatment with 400 μg/mL of UPE_8 resulted in the highest cell viability (83.15%). Finally, we confirmed the effect of treatment with UPE on ROS production in Vero cells after exposure to H_2_O_2_ (Figure 3C). Compared to the control, the amount of ROS produced after exposure to H_2_O_2_ significantly increased by 393.17% (*p* < 0.05), and this increase was inhibited by treatment with UPEs. UPE_2, UPE_4, UPE_6, and UPE_8 showed excellent ROS production inhibitory effects (148.71, 149.98, 150.92, and 149.89%, respectively), and no significant differences were observed between the extracts (*p* > 0.05) in Vero cells exposed to H_2_O_2_.

Oxidative stress-induced ROS overproduction is detrimental because it causes oxidative damage to cellular macromolecules [3]. ROS also induces apoptosis, affecting cell viability. In this study, the UPEs showed an inhibitory effect on H_2_O_2_-induced ROS production (Figure 3C). The decrease in ROS levels, mediated by the protective effect conferred by the UPEs, also affected Vero cell viability (Figure 3B). Similar results have also been reported; the extracts and fucoidan fractions obtained from brown seaweed (*Sargassum autumnale*) via enzyme-assisted extraction significantly reduce ROS production in Vero cells following exposure to H_2_O_2,_ and they increase cell viability [27]. Moreover, these extracts and fucoidan fractions also alleviate the H_2_O_2_-induced decrease in cell viability.

Considering the extraction yields, contents of polysaccharides, polyphenols, and fucoxanthin, and cell protective effects according to the extraction time, UPE_8 was superior; therefore, the subsequent experiments were conducted using only UPE_8. UPE_8 was further separated into UPE_8S and UPE_8P by using ethanol precipitation to identify the components contributing to the antioxidant activity of UPE_8 and confirm its antioxidant activity.

### 3.3. Polysaccharide Contents, Molecular Weight, and Monosaccharide Composition Analysis of UPE_8s

The polysaccharide content of UPE_8s is shown in Table 1. Before ethanol precipitation, the polysaccharide content of UPE_8 was 67.52 ± 1.94%. When ethanol precipitation was performed to separate crude polysaccharides, UPE_8P showed a significantly high polysaccharide content (80.29 ± 5.14%, *p* < 0.05). However, a polysaccharide content of 53.06 ± 3.72% was also measured in UPE_8S. The higher the ethanol concentration, the lower the molecular weight of the polysaccharide precipitated during ethanol precipitation [32]. In our study, the final concentration of ethanol was 33%, which was relatively low, and it is possible that the low-molecular-weight polysaccharides did not precipitate and were present in the supernatant. The molecular weight analysis results also confirmed this finding.

The results of the molecular weight analysis of UPE_8s are presented in Table 2. Two prominent peaks were detected for each sample. For UPE_8, the peak with the highest molecular weight had a molecular weight of 1062 kDa and a content of 30.27%. The 110 Da peak occupied 69.73% of the total area. In contrast, when ethanol precipitation was performed, high-molecular-weight polysaccharides were precipitated, as confirmed by UPE_8P analysis, and the ratio of the peak with the molecular weight of 745 kDa was 94.89%. For UPE_8S, most of the components (96.33%) had a molecular weight of 175 Da, confirming that polysaccharides were well separated by ethanol.

The biofunctional activities of polysaccharides are greatly influenced by the composition of their constituent monosaccharides [33]. Therefore, we performed a monosaccharide composition analysis of UPE_8s (Table 3). UPE_8 contained four monosaccharides (fucose, galactose, glucose, and xylose). Galactose was the major monosaccharide, accounting for 55.64% (22 μg/mg of dry weight extract) of the total polysaccharides content, followed by fucose with 18 μg/mg of dry weight extract, accounting for 31.90%. The fractions obtained using ethanol precipitation confirmed that the monosaccharide content was greatly reduced in UPE_8S, whereas it was increased in UPE_8P. These results suggest that ethanol precipitation effectively separates the polysaccharides present in UPE_8. Similarly to UPE_8, UPE_8P contained high levels of galactose (40 μg/mg of dry weight extract) and fucose (31 μg/mg of dry weight extract). Galactose and fucose are well-known monosaccharides with high antioxidant activity [34] and are regarded as key contributors to the antioxidant activity of UPE_8 (Figure 3).

### 3.4. Effects of UPEs on Cell Viability and H_2_O_2_-Induced ROS Production in Vero Cells

The cell protective effect and effect on ROS production of UPE_8S and UPE_8P were confirmed using H_2_O_2_-damaged Vero cells to confirm their antioxidant activity.

**Figure 4:**
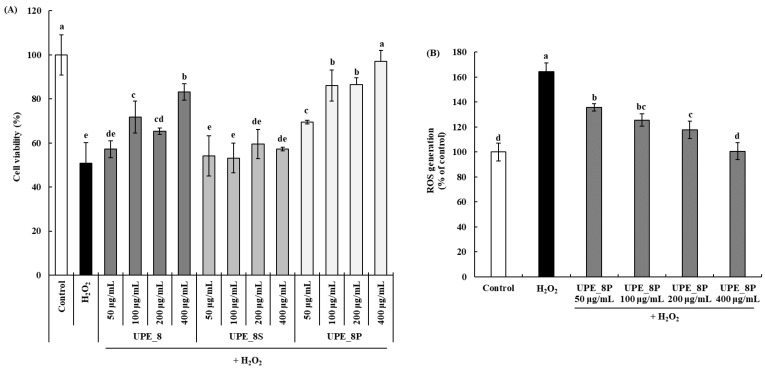
Effects of UPE_8S and UPE_8P on the protective effect (**A**) and ROS generation (**B**) of Vero cells induced with 1 mM H_2_O_2_. Values are expressed as mean ± standard deviation. Different letters (a–e) indicate significant differences found using one-way ANOVA followed by Duncan’s multiple range test (*p* < 0.05). Control: medium-only treated group.

The cell protective effects are shown in Figure 4A. Cell viability decreased after treatment with 1 mM H_2_O_2_ in a dose-dependent manner. A cell viability of 97.17% was observed at a concentration of 400 μg/mL UPE_8P, showing a significantly higher cell protective effect than UPE_8 (*p* < 0.05). In addition, UPE_8P showed a higher cell protective effect than UPE_8 at all concentrations (50–400 μg/mL, *p* < 0.05). In contrast, UPE_8S did not protect against cytotoxicity caused by H_2_O_2_ treatment at all concentrations. No significant difference in cell viability between the 1 mM H_2_O_2_-treated group and the UPE_8S-treated group (*p* > 0.05) was observed.

UPE_8P had a higher polysaccharide content than UPE_8S (Table 1). The high-molecular-weight polysaccharide content was significantly higher than that of UPE_8S (Table 2). Therefore, the high-molecular-weight polysaccharide components present in the precipitate could have excellent antioxidant activity and effectively protect Vero cells from cytotoxicity caused by H_2_O_2_-induced oxidative stress. Thus, the antioxidant activity of UPE_8 was derived from the polysaccharide component (specifically, the high-molecular-weight polysaccharide) present in *U. pinnatifida* sporophyll. Red (*Kappaphycus alvarezii*), green (*Kappaphycus striatus*), and brown (*Padina gymnospora*) marine seaweeds possess antioxidant activities [35], and the antioxidant activity of their extracts is derived from polysaccharides [35,36]. These antioxidant activities differed depending on the type of seaweed, and the higher the sugar content of the seaweed extract, the higher the 2,2-diphenyl-1-picrylhydrazyl and 2,2′-azino-bis(3-ethylbenzothiazoline-6-sulfonic acid) radical scavenging activities. Therefore, the importance of the relationship between polysaccharide content and the antioxidant activity of seaweeds has been reported. In addition, polysaccharides are a major component of seaweed extracts, and fucoidan is a representative sulfated polysaccharide present in the cell walls of seaweeds [36]. Fucoidan isolated from Malaysian seaweeds can be commercialized as a natural antioxidant [36]. Therefore, the antioxidant activity of the seaweed extracts was likely due to the antioxidant activity of fucoidan. Fucoidans contain a substantial proportion of fucose and sulfate ester groups [37]. In our study, the monosaccharide composition of UPE_8P confirmed that its fucose content was high. Collectively, these results indicate that the high antioxidant activity of UPE_8P is attributable to its high fucoidan content.

We further confirmed the effect of UPE_8P, which showed excellent cytotoxicity protection in Vero cells, on H_2_O_2_-induced ROS production (Figure 4B). Following exposure to H_2_O_2_, ROS production cells increased in Vero up to 164.18% compared to the control cell. However, following treatment with UPE_8P, ROS levels decreased in a dose-dependent manner (*p* < 0.05). Even at the highest concentration (400 μg/mL), ROS production was effectively suppressed to the level observed in the control group. Therefore, the effective suppression by UPE_8P of ROS induced by oxidative stress may be related to its protective effects against cytotoxicity (Figure 4A).

### 3.5. Effects of UPE_8P on the Expression of Apoptosis-Related Proteins in Vero Cells

We further analyzed the effect of UPE_8P on the expression of apoptosis-related proteins in H_2_O_2_-exposed Vero cells using Western blotting. The Bcl-2 protein family is a protein associated with cellular apoptosis [38] and can be divided into two groups: pro-apoptotic (Bax, Bok, and Bad) and anti-apoptotic (Bcl-2, Bcl-XL, and Mcl-1). Many studies have assessed the effect of a given treatment on the expression of pro-apoptotic and anti-apoptotic proteins as a means to evaluate its effect on cellular damage caused by oxidative stress [3,39,40].

**Figure 5:**
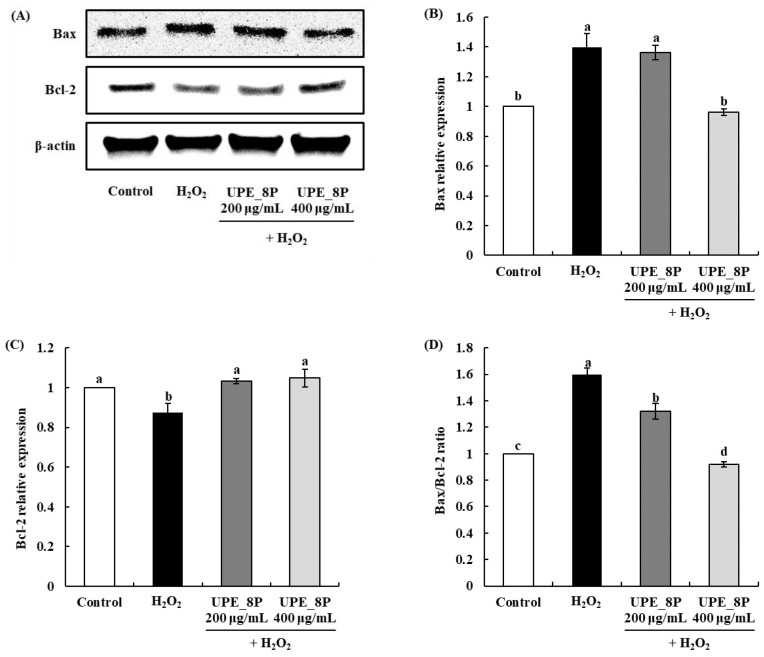
Effects of UPE_8P on Bax and Bcl-2 protein expressions (**A**–**C**) and Bax/Bcl-2 ratio (**D**) of Vero cells induced with 1 mM H_2_O_2_. Values are expressed as mean ± standard deviation. Different letters (a–d) among samples indicate significant differences found using one-way ANOVA followed by Duncan’s multiple range test (*p* < 0.05). Control: medium-only treated group.

The expression of Bax, a pro-apoptotic protein, was significantly increased compared to that in the control when H_2_O_2_ was used as the oxidative stress inducer (*p* < 0.05; Figure 5). However, upon treatment with UPE_8P, a significant decrease in Bax expression was observed (400 μg/mL, *p* < 0.05). In addition, the expression of Bcl-2, an anti-apoptotic protein, was significantly decreased upon exposure to H_2_O_2_ compared to that in the control (*p* < 0.05). However, treatment with UPE_8P at all concentrations rescued the expression of Bcl-2 to a level similar to that in control cells (*p* < 0.05). This result suggests that UPE_8P can effectively inhibit the apoptosis of cells caused by H_2_O_2_. Finally, the Bax/Bcl-2 ratio, which is a marker of apoptosis, increased upon exposure to H_2_O_2_ but decreased in a dose-dependent manner when treated with UPE_8P (Figure 5D, *p* < 0.05).

Many studies have reported the effects of antioxidants on the expression of apoptosis-related proteins. Polysaccharides extracted from *Pleurotus ostreatus* with strong antioxidant activity exhibited protective effects against H_2_O_2_-induced apoptosis in PC12 cells [39], and the increased Bax/Bcl-2 ratio was effectively decreased after treatments with polysaccharide. Furthermore, edible insect protein isolates exhibit protective effects against cytotoxicity by regulating the expression of apoptosis-related proteins; those effects are conferred by the potent antioxidant activity of these isolates [3]. Therefore, in the present study, the effect of UPE_8P on Vero cell apoptosis was attributed to its strong antioxidant activity.

Various types of seaweed polysaccharides exhibit antioxidant activities [17,18,41,42,43]. Premarathna et al. [41] extracted polysaccharides from five types of seaweed and reported their antioxidant activity using various types of assays. Among these, the SOD assay results highlighted the importance of antioxidant activity in protecting cellular function from oxidative stress. In the present study, the antioxidant activity of seaweed extract was confirmed in a Vero cell model, and its effect on the expression of apoptosis-related proteins was demonstrated. In addition, because the method of extracting active ingredients such as polysaccharides from seaweed is crucial, various methods have been investigated in previous studies. These extraction methods include using organic solvents [17] and various enzymes [18]. Although the extracts obtained using such extraction methods showed high antioxidant activity, their practical industrial use presented challenges, including the need for a process that requires the complete removal of organic solvents and low economic feasibility due to expensive enzymes. The ultrasonication extraction method using water presented in our study is a promising extraction method that can overcome these issues. Notably, the ease of upscaling ultrasonication for industrialization has been previously demonstrated [24]. Moreover, a recent study has reported the cosmetic application potential of polysaccharide, which is extracted from seaweed and has strong antioxidant activity [42]. The study reported that the extracted polysaccharides effectively inhibit the activity of elastase, an enzyme related to skin health. Therefore, the potential for the industrial use of extracts obtained using ultrasonication extraction may be expanded from the food industry to the cosmetics industry through further research.

## 4. Conclusions

UPEs, extracted from *U. pinnatifida* sporophylls using ultrasonication, exhibited antioxidant and protective effects against H_2_O_2_-induced oxidative stress in Vero cells. The extraction yield, polysaccharide content, and antioxidant activity of the samples were measured at different extraction times. UPE_8 had the highest extraction yield and polysaccharide content, exhibited excellent cell protective effects, and inhibited H_2_O_2_-induced ROS production in Vero cells. Notably, the polysaccharide content and molecular weight analysis of UPE_8 revealed a high polysaccharide content, and most of the high-molecular-weight polysaccharides were detected in UPE_8P after precipitation with ethanol. These results were confirmed by the monosaccharide component analysis. Moreover, UPE_8P had a strong protective effect against oxidative stress (H_2_O_2_ treatment) in Vero cells, whereas UPE_8S showed no protective effect. Thus, the antioxidant activity of UPE_8 is likely attributable to its polysaccharide content, particularly fucose and galactose. Furthermore, UPE_8P effectively inhibited the apoptosis of Vero cells following H_2_O_2_ treatment. These findings suggest that the UPE obtained by ultrasonication can be used as a functional food ingredient in the food industry with excellent antioxidant activity.

## Figures and Tables

**Table 1:** Polysaccharide contents of UPE_8s.

	UPE_8	UPE_8S	UPE_8P
Polysaccharide contents (%)	67.52 ± 1.94 ^b^	53.08 ± 3.72 ^c^	80.29 ± 5.14 ^a^

Values are expressed as mean ± standard deviation. Different letters (^a–c^) indicate significant differences found using one-way ANOVA followed by Duncan’s multiple range test (*p* < 0.05).

**Table 2:** Molecular weight analysis of UPE_8s.

		UPE_8	UPE_8S	UPE_8P
Molecular weight (Da, (%))	Peak 1	1,062,710 (30.27)	447,298 (3.67)	745,555 (94.89)
	Peak 2	110 (69.73)	175 (96.33)	143 (5.11)

**Table 3:** Monosaccharide composition analysis of UPE_8s.

		UPE_8	UPE_8S	UPE_8P
Monosaccharide composition (μg/mg of dry weight extract, (%))	Fucose	18 (31.90)	5 (29.45)	31 (32.33)
	Rhamnose	nd	nd	1 (0.35)
	Arabinose	nd	nd	nd
	Galactose	22 (55.64)	5 (34.93)	40 (59.39)
	Glucose	3 (7.30)	3 (21.97)	3 (4.54)
	Xylose	1 (3.32)	1 (9.08)	2 (2.98)
	Fructose	nd	1 (2.52)	nd

nd: not determined.

## Data Availability

No new data were created or analyzed in this study. Data sharing is not applicable to this article.
